# Supplementary figures and images for: Estimating the impact of differential adherence on the comparative effectiveness of stool-based colorectal cancer screening using the CRC-AIM microsimulation model
Source: PLoS One. 2020 Dec 29;15(12):e0244431. doi: 10.1371/journal.pone.0244431 (PMC7771985; doi:10.1371/journal.pone.0244431)

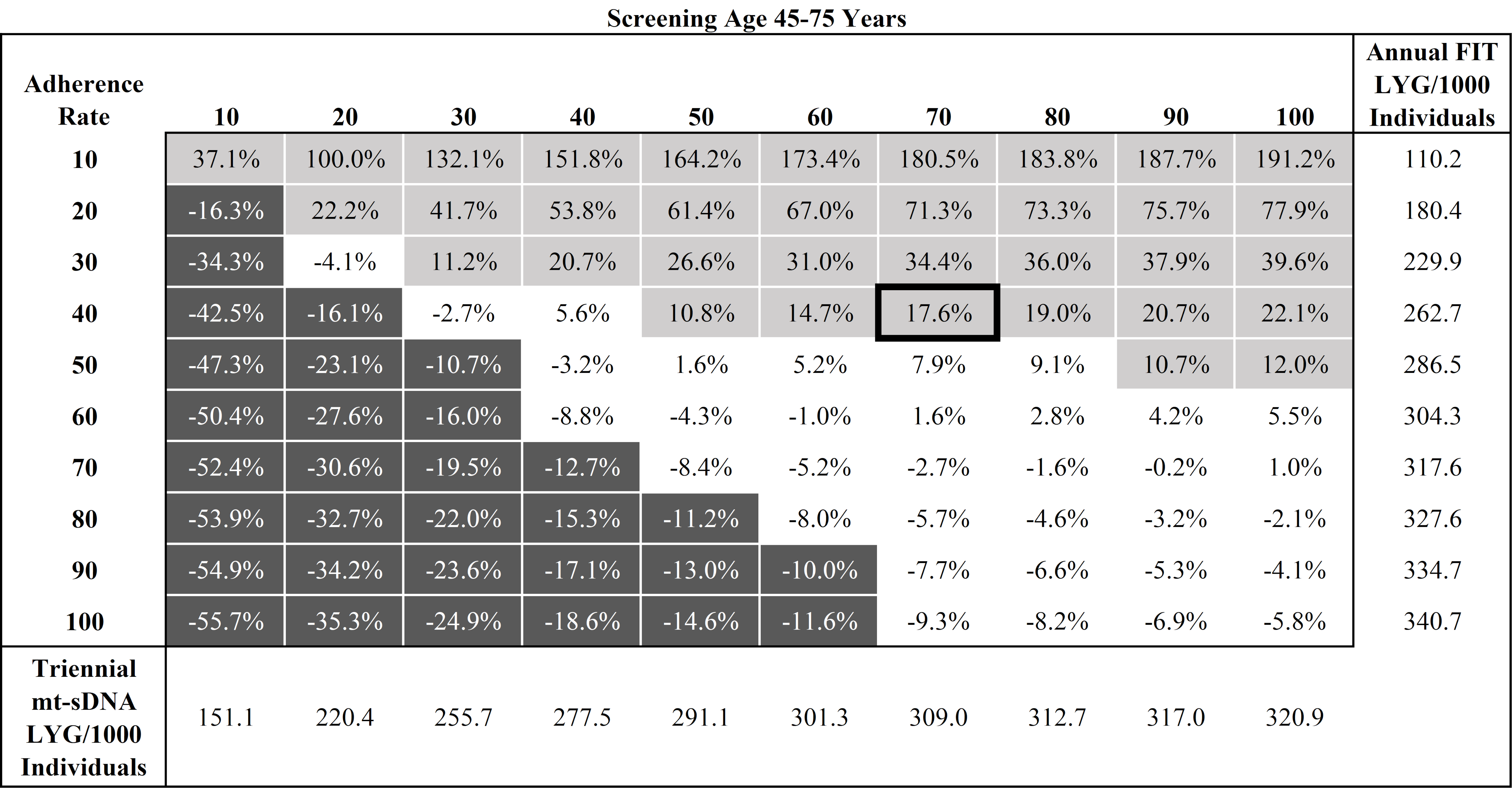

Supplement: S1 Fig — White boxes indicate <10% difference between tests. Light gray boxes indicate ≥10% positive difference with mt-sDNA versus FIT. Dark gray boxes indicate ≥10% negative difference with mt-sDNA versus FIT. Outlined box indicates base-case imperfect adherence rates. (TIF) [file pone.0244431.s001.tif]

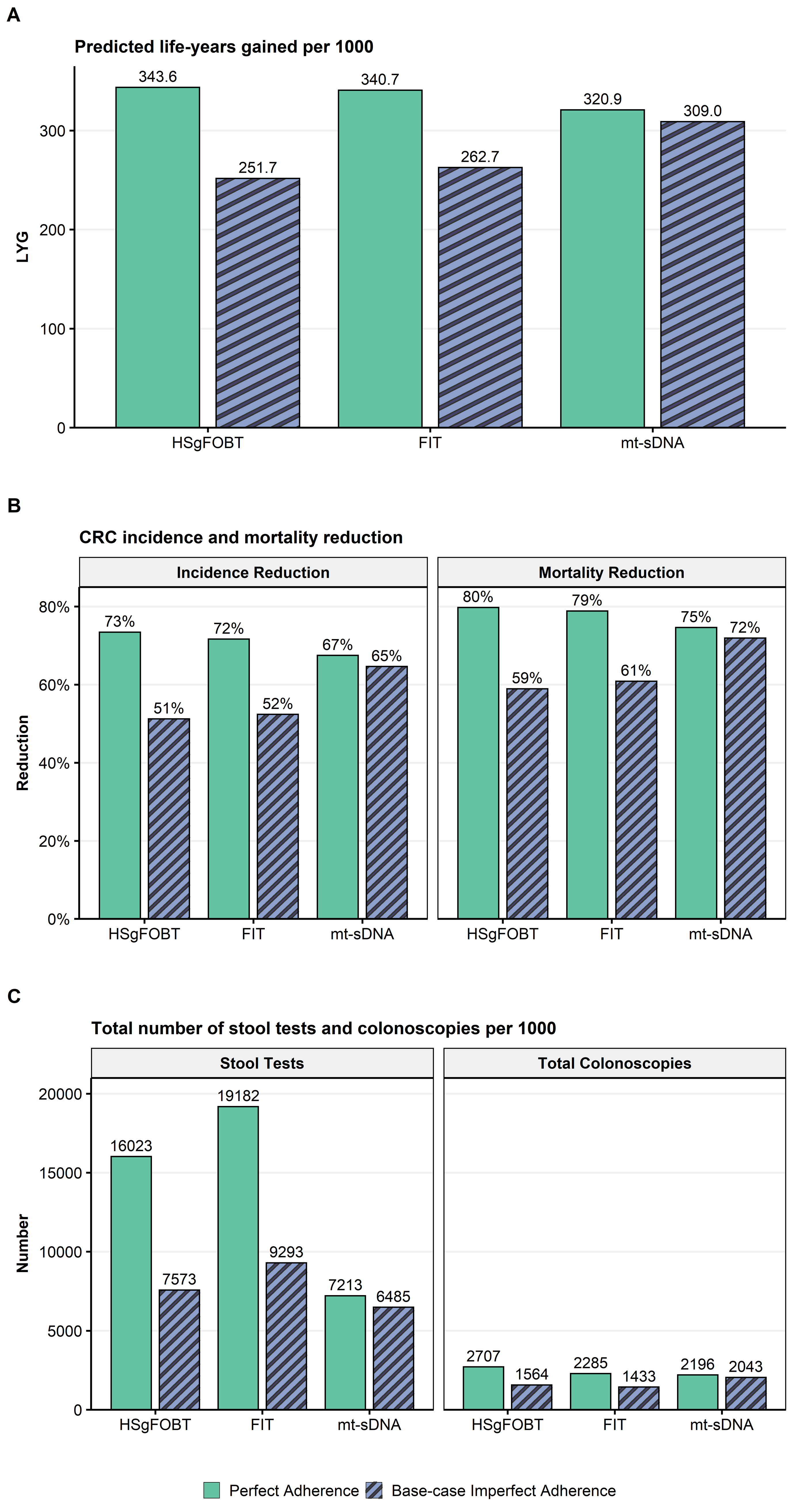

Supplement: S2 Fig — A) Predicted life-years gained (LYG), B) CRC-related incidence and mortality reduction, and C) total stool tests and colonoscopies (COL) per 1000 individuals screened from ages 45–75 compared with no screening assuming perfect (100%) adherence rates to annual FIT, annual HSgFOBT, and triennial mt-sDNA or base-case imperfect adherence (40% FIT vs 34% HSgFOBT vs 70% mt-sDNA). (TIF) [file pone.0244431.s002.tif]

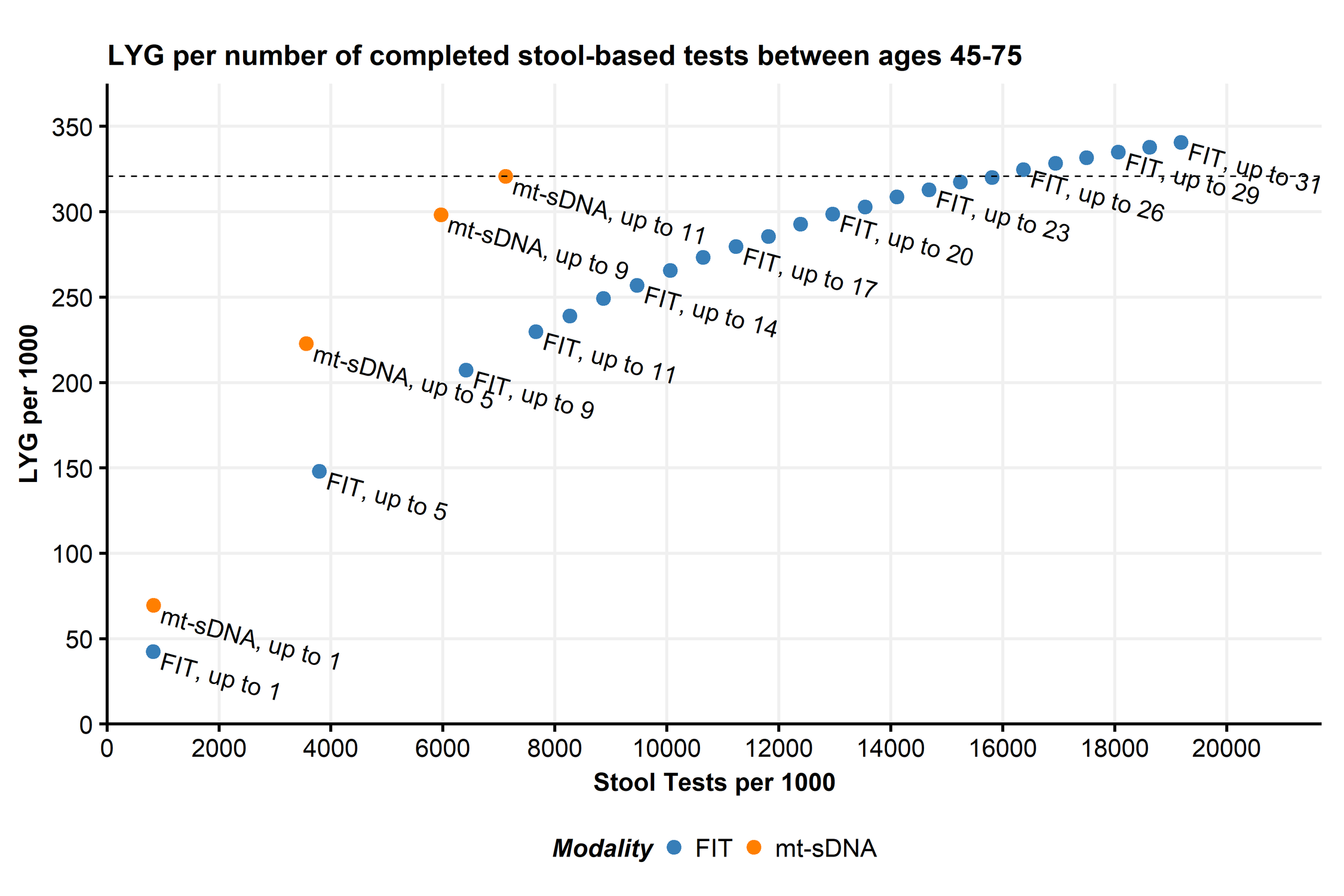

Supplement: S3 Fig — Individuals were randomly assigned numbers of multitarget stool DNA (mt-sDNA; max = 11 triennial tests during the screening window) or fecal immunochemical tests (FIT; max = 31 annual tests during the screening window). The line indicates equivalent LYG with up to 11 mt-sDNA tests. (TIF) [file pone.0244431.s003.tif]

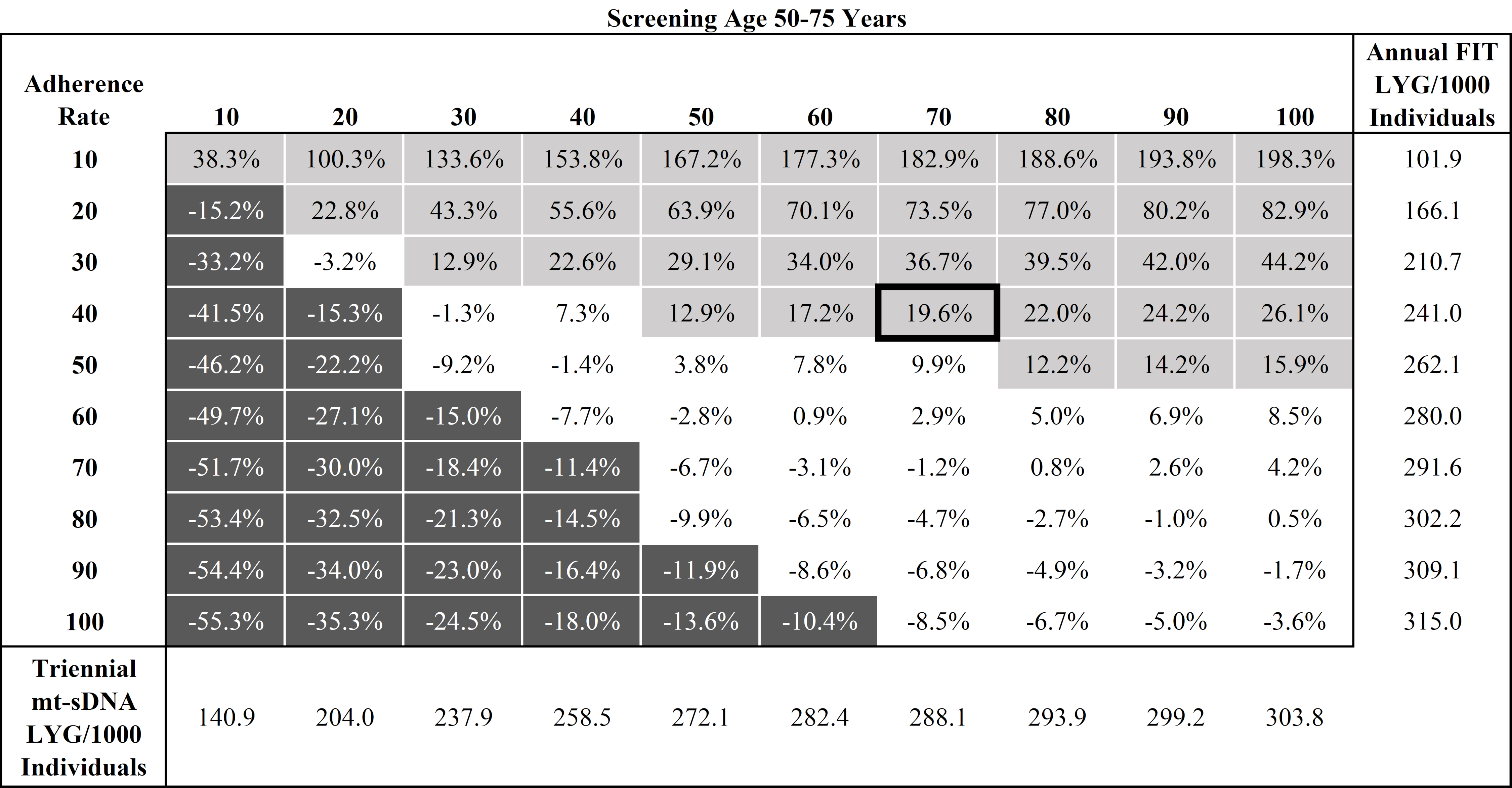

Supplement: S4 Fig — White boxes indicate <10% difference between tests. Light gray boxes indicate ≥10% positive difference with mt-sDNA versus FIT. Dark gray boxes indicate ≥10% negative difference with mt-sDNA versus FIT. Outlined box indicates base-case imperfect adherence rates. (TIF) [file pone.0244431.s004.tif]

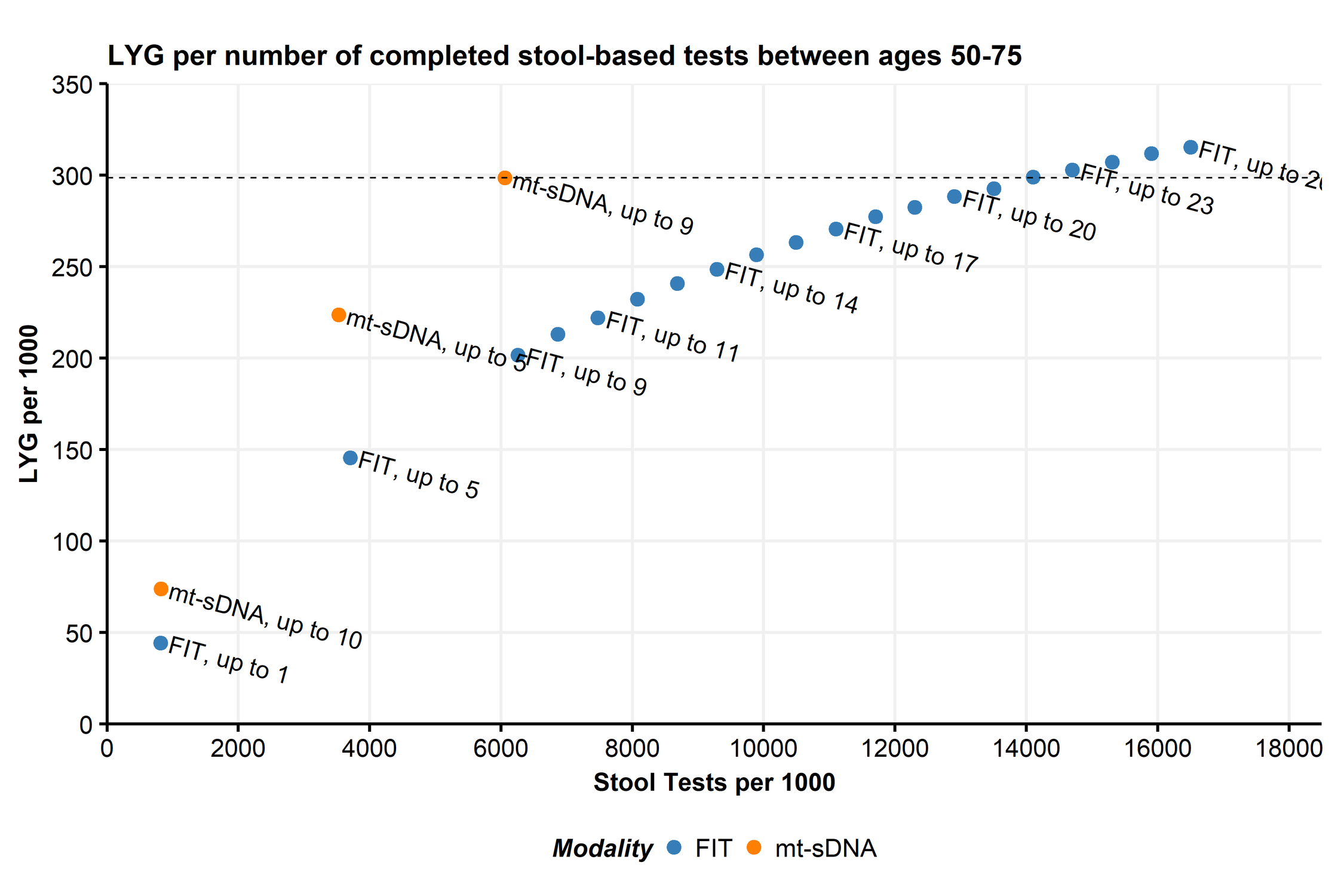

Supplement: S5 Fig — Individuals were randomly assigned numbers of multitarget stool DNA (mt-sDNA; max = 9 triennial tests during the screening window) or fecal immunochemical tests (FIT; max = 26 annual tests during the screening window). The line indicates equivalent LYG with up to 9 mt-sDNA tests. (TIF) [file pone.0244431.s005.tif]

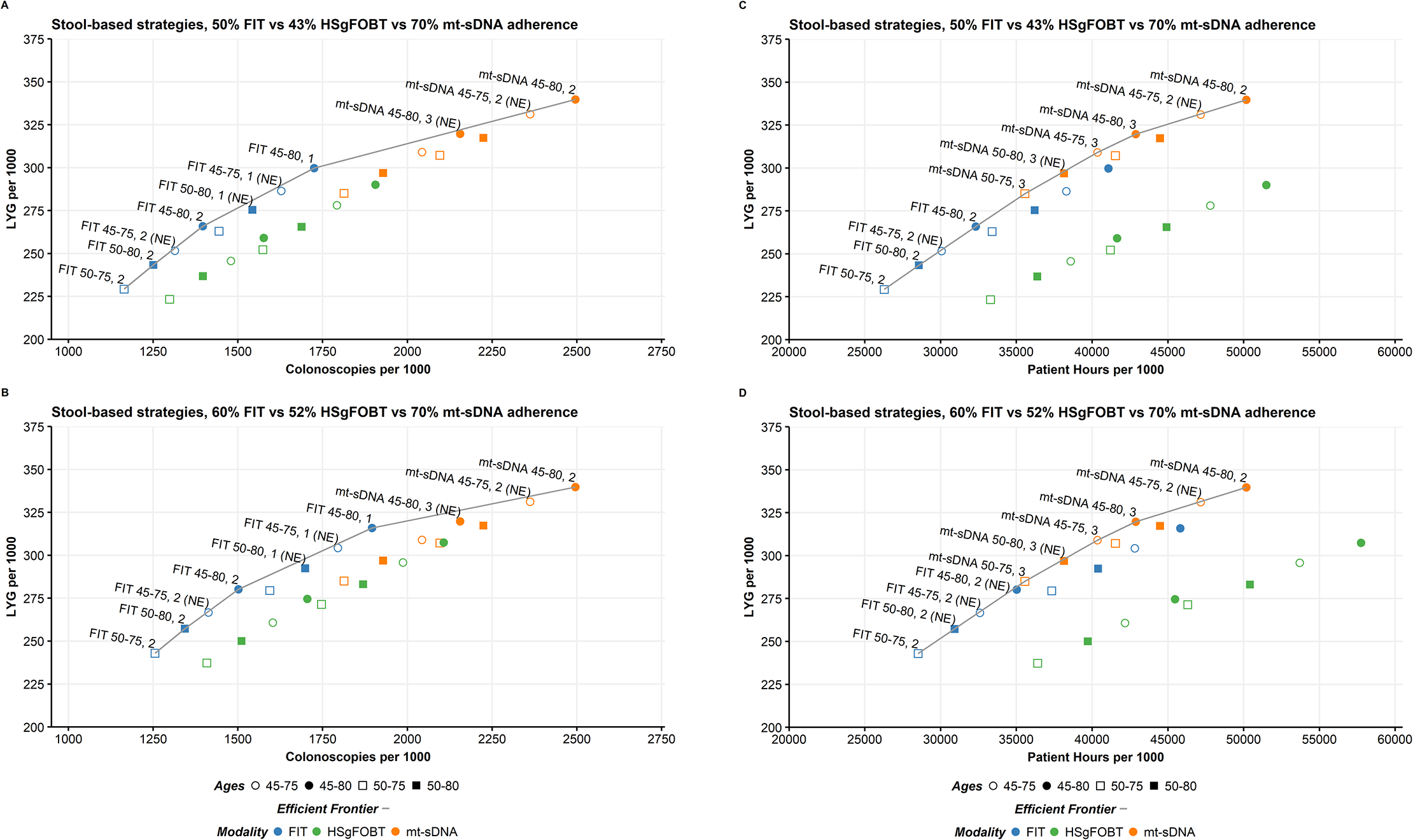

Supplement: S6 Fig — Sensitivity analysis of life-years gained for individuals 40 years of age with stool-based tests A) number of colonoscopies assuming 50% FIT vs 43% HSgFOBT vs 70% mt-sDNA adherence or B) assuming 60% FIT vs 52% HSgFOBT vs 70% mt-sDNA adherence and by C) patient hours related to the screening process and assuming 50% FIT vs 43% HSgFOBT vs 70% mt-sDNA adherence or D) assuming 60% FIT vs 52% HSgFOBT vs 70% mt-sDNA adherence. Results shown are per 1000 individuals free of diagnosed colorectal cancer at age 40 and screened starting at age 45 or 50 and ending at age 75 or 80 receiving biennial or triennial mt-sDNA, annual or biennial FIT, and annual or biennial HSgFOBT. NE, near-efficient. (TIF) [file pone.0244431.s006.tif]

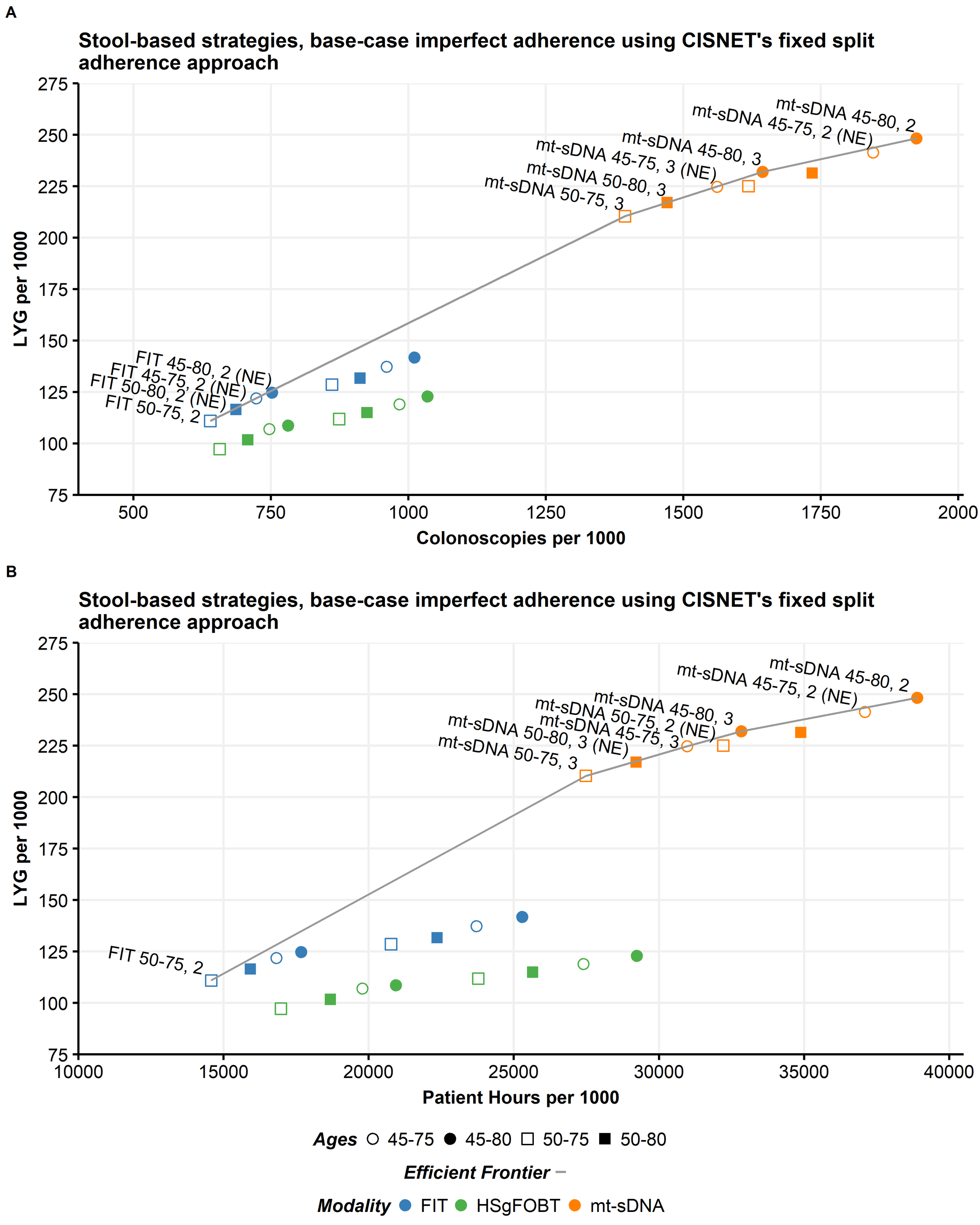

Supplement: S7 Fig — Life-years gained for individuals 40 years of age with stool-based tests by A) number of colonoscopies or B) patient hours related to the screening process assuming base-case imperfect adherence rates of 40% FIT vs 34% HSgFOBT vs 70% mt-sDNA using the CISNET fixed split adherence approach. Results shown are per 1000 individuals free of diagnosed colorectal cancer at age 40 and screened starting at age 45 or 50 and ending at age 75 or 80 receiving biennial or triennial mt-sDNA, annual or biennial FIT, and annual or biennial HSgFOBT. NE, near efficient. (TIF) [file pone.0244431.s007.tif]
