# Supplementary material for: Estimating the impact of differential adherence on the comparative effectiveness of stool-based colorectal cancer screening using the CRC-AIM microsimulation model
Source: PLoS One. 2020 Dec 29;15(12):e0244431. doi: 10.1371/journal.pone.0244431 (PMC7771985; doi:10.1371/journal.pone.0244431)
Supplement: S1 Table — (DOCX) [file pone.0244431.s008.docx]

**S1 Table. Screening characteristics for Deep-C (clinicaltrials.gov identifier, NCT01397747) sensitivity analyses using granular adenoma size.**

|  | **Sensitivity** | | | | **Specificity** | | | | |
| --- | --- | --- | --- | --- | --- | --- | --- | --- | --- |
|  | **Adenomas, mm** | | | **Cancer Stage** | **Age, y** | | | | |
|  | **<6** | **6**–**9** | **≥10** | **I**–I**V** | **<60** | **60**–**64** | **65**–**69** | **70**–**74** | **75+** |
| FIT, % (n/N) | 6.0%  (117/1944) | 11.1%  (113/1015) | 24.6%  (170/691) | 73.8%  (48/65) | 97.8%  (1361/1392) | 96.5%  (355/368) | 95.6%  (1483/1551) | 96.7%  (697/721) | 93.9%  (399/425) |
| 95% CI | 5.0% – 7.2% | 9.3% – 13.2% | 21.4% – 28.0% | 61.5% – 84.0% | 96.9% – 98.5% | 94.0% – 98.1% | 94.5% – 96.6% | 95.1% – 97.9% | 91.2% – 96.0% |
| mt-sDNA, % (n/N) | 14.9%  (290/1944) | 22.5%  (228/1015) | 43.6%  (301/691) | 92.3%  (60/65) | 94.4%  (1314/1392) | 92.4%  (340/368) | 89.1%  (1382/1551) | 86.1%  (621/721) | 81.2%  (345/425) |
| 95% CI | 13.4% – 16.6% | 19.9% – 25.2% | 39.8% –47.4% | 83.0% – 97.5% | 93.1% – 95.5% | 89.2% – 94.9% | 87.4% – 90.6% | 83.4% – 88.6% | 77.1% – 84.8% |

FIT, fecal immunochemical test; mt-sDNA, multitarget stool DNA test.

2-sided 95% confidence intervals calculated using the Exact (Clopper-Pearson) method.
