# Supplementary material for: Estimating the impact of differential adherence on the comparative effectiveness of stool-based colorectal cancer screening using the CRC-AIM microsimulation model
Source: PLoS One. 2020 Dec 29;15(12):e0244431. doi: 10.1371/journal.pone.0244431 (PMC7771985; doi:10.1371/journal.pone.0244431)
Supplement: S2 Table — (DOCX) [file pone.0244431.s009.docx]

**S2 Table.** **Screening outcomes per 1000 individuals by adherence rate for triennial mt-sDNA, annual FIT, and annual HSgFOBT in individuals free of diagnosed colorectal cancer at age 40 and screened between ages 50–75 years or 45–75 years.**

| **Screening Strategy and Adherence Rate** | **Stool Tests** | **Follow-up COLs** | **Surveillance  COLs** | **Total  COLs** | **CRC  Cases** | **CRC  Deaths** | **LY with CRC** | **LYG** | **Incidence Reduction** | **Mortality Reduction** |
| --- | --- | --- | --- | --- | --- | --- | --- | --- | --- | --- |
| No screening | 0 | 0 | 0 | 80 | 80.3 | 36.6 | 646.0 | 0.0 | 0.0% | 0.0% |
| mt-sDNA 50-75, 3 |  |  |  |  |  |  |  |  |  |  |
| 10% | 1,782 | 268 | 452 | 770 | 57.6 | 24.6 | 526.9 | 133.1 | 28.3% | 33.0% |
| 20% | 2,925 | 424 | 684 | 1,145 | 46.8 | 18.9 | 464.0 | 196.0 | 41.7% | 48.3% |
| 30% | 3,717 | 527 | 825 | 1,382 | 40.6 | 15.9 | 422.0 | 231.1 | 49.4% | 56.5% |
| 40% | 4,304 | 601 | 915 | 1,541 | 36.9 | 14.0 | 397.0 | 253.8 | 54.1% | 61.7% |
| 50% | 4,751 | 657 | 978 | 1,659 | 34.4 | 12.8 | 378.5 | 268.1 | 57.2% | 65.0% |
| 60% | 5,107 | 701 | 1,024 | 1,747 | 32.6 | 12.0 | 364.0 | 278.3 | 59.4% | 67.2% |
| 70% | 5,397 | 734 | 1,059 | 1,813 | 31.3 | 11.4 | 353.4 | 285.1 | 61.1% | 68.8% |
| 80% | 5,641 | 763 | 1,085 | 1,867 | 30.4 | 11.0 | 346.9 | 290.2 | 62.2% | 70.0% |
| 90% | 5,864 | 789 | 1,108 | 1,916 | 29.4 | 10.6 | 339.5 | 295.3 | 63.4% | 71.2% |
| 100% | 6,076 | 814 | 1,126 | 1,957 | 28.6 | 10.2 | 333.0 | 300.0 | 64.4% | 72.2% |
| FIT 50-75, 1 |  |  |  |  |  |  |  |  |  |  |
| 10% | 2,174 | 142 | 285 | 486 | 65.5 | 28.1 | 582.6 | 96.3 | 18.5% | 23.2% |
| 20% | 4,162 | 258 | 500 | 802 | 55.1 | 22.4 | 530.7 | 160.2 | 31.5% | 38.8% |
| 30% | 5,980 | 356 | 666 | 1,057 | 47.2 | 18.4 | 483.8 | 206.5 | 41.3% | 49.8% |
| 40% | 7,672 | 441 | 799 | 1,269 | 41.3 | 15.5 | 444.4 | 239.4 | 48.6% | 57.6% |
| 50% | 9,246 | 518 | 903 | 1,444 | 36.9 | 13.5 | 411.5 | 263.0 | 54.1% | 63.1% |
| 60% | 10,727 | 585 | 987 | 1,594 | 33.6 | 12.0 | 384.9 | 279.5 | 58.2% | 67.2% |
| 70% | 12,120 | 648 | 1,060 | 1,726 | 30.8 | 10.9 | 360.5 | 293.0 | 61.6% | 70.3% |
| 80% | 13,432 | 705 | 1,120 | 1,842 | 28.7 | 9.9 | 342.8 | 304.4 | 64.3% | 72.9% |
| 90% | 14,673 | 759 | 1,170 | 1,945 | 26.9 | 9.3 | 323.7 | 310.9 | 66.5% | 74.6% |
| 100% | 15,856 | 808 | 1,214 | 2,037 | 25.4 | 8.7 | 308.9 | 318.1 | 68.4% | 76.3% |
| HSgFOBT 50-75, 1 |  |  |  |  |  |  |  |  |  |  |
| 10% | 2,127 | 208 | 306 | 572 | 64.4 | 27.7 | 572.4 | 99.8 | 19.9% | 24.4% |
| 20% | 3,981 | 379 | 532 | 954 | 53.1 | 21.7 | 511.5 | 166.3 | 33.8% | 40.6% |
| 30% | 5,612 | 520 | 701 | 1,255 | 45.3 | 17.8 | 463.8 | 211.9 | 43.7% | 51.5% |
| 40% | 7,074 | 642 | 833 | 1,503 | 39.3 | 14.9 | 422.0 | 244.2 | 51.1% | 59.3% |
| 50% | 8,373 | 752 | 934 | 1,709 | 34.9 | 12.9 | 390.0 | 268.0 | 56.5% | 64.9% |
| 60% | 9,558 | 846 | 1,016 | 1,883 | 31.7 | 11.5 | 361.5 | 284.9 | 60.6% | 68.7% |
| 70% | 10,631 | 933 | 1,084 | 2,034 | 29.1 | 10.4 | 338.2 | 296.6 | 63.7% | 71.6% |
| 80% | 11,609 | 1,011 | 1,139 | 2,167 | 27.1 | 9.5 | 321.6 | 307.3 | 66.3% | 74.0% |
| 90% | 12,505 | 1,081 | 1,188 | 2,285 | 25.4 | 8.9 | 302.8 | 314.2 | 68.4% | 75.7% |
| 100% | 13,337 | 1,146 | 1,227 | 2,388 | 24.0 | 8.4 | 288.4 | 320.6 | 70.1% | 77.0% |
| mt-sDNA 45-75, 3 |  |  |  |  |  |  |  |  |  |  |
| 10% | 2,155 | 309 | 523 | 880 | 55.0 | 23.3 | 499.9 | 151.1 | 31.5% | 36.3% |
| 20% | 3,522 | 491 | 781 | 1,306 | 43.6 | 17.5 | 426.7 | 220.4 | 45.8% | 52.2% |
| 30% | 4,472 | 609 | 929 | 1,565 | 37.4 | 14.6 | 381.5 | 255.7 | 53.4% | 60.3% |
| 40% | 5,171 | 696 | 1,022 | 1,742 | 33.9 | 12.8 | 355.2 | 277.5 | 57.8% | 65.1% |
| 50% | 5,711 | 759 | 1,089 | 1,869 | 31.4 | 11.7 | 334.6 | 291.1 | 60.9% | 68.1% |
| 60% | 6,138 | 809 | 1,138 | 1,967 | 29.7 | 10.9 | 318.8 | 301.3 | 63.1% | 70.3% |
| 70% | 6,485 | 851 | 1,174 | 2,043 | 28.4 | 10.3 | 308.8 | 309.0 | 64.6% | 71.9% |
| 80% | 6,771 | 883 | 1,202 | 2,103 | 27.5 | 9.9 | 298.9 | 312.7 | 65.8% | 72.9% |
| 90% | 6,995 | 911 | 1,224 | 2,151 | 26.8 | 9.6 | 292.6 | 317.0 | 66.7% | 73.8% |
| 100% | 7,213 | 935 | 1,245 | 2,196 | 26.1 | 9.3 | 287.4 | 320.9 | 67.5% | 74.6% |
| FIT 45-75, 1 |  |  |  |  |  |  |  |  |  |  |
| 10% | 2,637 | 162 | 331 | 550 | 63.7 | 27.3 | 565.1 | 110.2 | 20.7% | 25.5% |
| 20% | 5,035 | 297 | 574 | 913 | 52.3 | 21.2 | 501.6 | 180.4 | 34.8% | 42.0% |
| 30% | 7,239 | 408 | 757 | 1,198 | 44.2 | 17.1 | 448.1 | 229.9 | 45.0% | 53.3% |
| 40% | 9,293 | 507 | 900 | 1,433 | 38.2 | 14.3 | 402.9 | 262.7 | 52.4% | 60.9% |
| 50% | 11,191 | 595 | 1,011 | 1,628 | 33.9 | 12.3 | 368.4 | 286.5 | 57.8% | 66.4% |
| 60% | 12,978 | 675 | 1,101 | 1,795 | 30.5 | 10.8 | 338.9 | 304.3 | 62.0% | 70.5% |
| 70% | 14,663 | 747 | 1,177 | 1,941 | 27.9 | 9.7 | 314.6 | 317.6 | 65.3% | 73.5% |
| 80% | 16,249 | 814 | 1,238 | 2,067 | 25.8 | 8.9 | 293.6 | 327.6 | 67.9% | 75.8% |
| 90% | 17,751 | 877 | 1,292 | 2,183 | 24.1 | 8.2 | 274.5 | 334.7 | 70.0% | 77.5% |
| 100% | 19,182 | 935 | 1,336 | 2,285 | 22.8 | 7.7 | 261.3 | 340.7 | 71.7% | 78.9% |
| HSgFOBT 45-75, 1 |  |  |  |  |  |  |  |  |  |  |
| 10% | 2,575 | 244 | 355 | 654 | 62.4 | 26.8 | 553.2 | 114.7 | 22.3% | 26.8% |
| 20% | 4,808 | 445 | 608 | 1,093 | 50.3 | 20.5 | 480.2 | 186.6 | 37.3% | 44.0% |
| 30% | 6,772 | 609 | 794 | 1,434 | 42.1 | 16.4 | 423.2 | 235.3 | 47.6% | 55.2% |
| 40% | 8,520 | 756 | 934 | 1,715 | 36.1 | 13.6 | 378.8 | 270.1 | 55.1% | 62.9% |
| 50% | 10,086 | 881 | 1,043 | 1,944 | 31.8 | 11.7 | 344.2 | 293.0 | 60.4% | 68.2% |
| 60% | 11,505 | 993 | 1,128 | 2,139 | 28.6 | 10.2 | 312.7 | 309.3 | 64.4% | 72.0% |
| 70% | 12,782 | 1,098 | 1,197 | 2,311 | 26.2 | 9.3 | 290.3 | 321.2 | 67.4% | 74.7% |
| 80% | 13,965 | 1,187 | 1,254 | 2,457 | 24.2 | 8.5 | 271.1 | 330.9 | 69.9% | 76.9% |
| 90% | 15,041 | 1,272 | 1,303 | 2,589 | 22.6 | 7.8 | 253.5 | 338.5 | 71.9% | 78.6% |
| 100% | 16,023 | 1,350 | 1,344 | 2,707 | 21.3 | 7.4 | 239.1 | 343.6 | 73.4% | 79.8% |

COL, colonoscopy; CRC, colorectal cancer; FIT, fecal immunochemical test; HSgFOBT, high-sensitivity guaiac-based fecal occult blood test; LY, life-years; LYG, life-years gained; mt-sDNA, multitarget stool DNA test.
