# Supplementary material for: Estimating the impact of differential adherence on the comparative effectiveness of stool-based colorectal cancer screening using the CRC-AIM microsimulation model
Source: PLoS One. 2020 Dec 29;15(12):e0244431. doi: 10.1371/journal.pone.0244431 (PMC7771985; doi:10.1371/journal.pone.0244431)
Supplement: S3 Table — Results are ordered by total colonoscopies. Results shown are per 1000 individuals free of diagnosed colorectal cancer at age 40 and screened starting at age 45 or 50 and ending at age 75 or 80 receiving biennial or triennial mt-sDNA, annual or biennial FIT, and annual or biennial HSgFOBT. Bold row is the model-recommended strategy. (DOCX) [file pone.0244431.s010.docx]

**S3 Table.** **Outcomes and efficiency ratios of LYG relative to number of colonoscopies assuming perfect (100%) adherence.** Results are ordered by total colonoscopies. Results shown are per 1000 individuals free of diagnosed colorectal cancer at age 40 and screened starting at age 45 or 50 and ending at age 75 or 80 receiving biennial or triennial mt-sDNA, annual or biennial FIT, and annual or biennial HSgFOBT. Bold row is the model-recommended strategy.

| **Screening Strategy** | **Stool Tests** | **Total COL** | **LYG** | **Complications** | **CRC Deaths Averted** | **ΔCOL** | **ΔLYG** | **Efficiency Ratio**  **(ΔCOL/ΔLYG)** | **Detail** |
| --- | --- | --- | --- | --- | --- | --- | --- | --- | --- |
| FIT 50-75, 2 | 9,447 | 1,480 | 273.5 | 9 | 23.7 | ND | ND | ND | Efficient |
| FIT 50-80, 2 | 10,790 | 1,598 | 289.3 | 11 | 26.0 | 118.0 | 15.7 | 7.5 | Efficient |
| FIT 45-75, 2 | 11,670 | 1,686 | 300.2 | 10 | 25.3 | 87.8 | 10.9 | 8.0 | Near Efficient |
| HSgFOBT 50-75, 2 | 8,627 | 1,758 | 278.5 | 10 | 24.3 | ND | ND | ND | Strongly Dominated |
| FIT 45-80, 2 | 12,562 | 1,763 | 310.9 | 11 | 26.8 | 165.1 | 21.6 | 7.6 | Efficient |
| HSgFOBT 50-80, 2 | 9,831 | 1,905 | 293.5 | 11 | 26.5 | ND | ND | ND | Strongly Dominated |
| mt-sDNA 50-75, 3 | 6,076 | 1,957 | 300.0 | 11 | 26.4 | ND | ND | ND | Strongly Dominated |
| HSgFOBT 45-75, 2 | 10,608 | 2,025 | 305.8 | 10 | 25.9 | ND | ND | ND | Strongly Dominated |
| FIT 50-75, 1 | 15,856 | 2,037 | 318.1 | 11 | 27.9 | ND | ND | ND | Weakly Dominated |
| mt-sDNA 50-80, 3 | 6,676 | 2,068 | 309.5 | 12 | 27.9 | ND | ND | ND | Strongly Dominated |
| HSgFOBT 45-80, 2 | 11,407 | 2,120 | 315.4 | 11 | 27.4 | ND | ND | ND | Strongly Dominated |
| FIT 50-80, 1 | 17,642 | 2,159 | 328.2 | 13 | 29.5 | ND | ND | ND | Weakly Dominated |
| mt-sDNA 45-75, 3 | 7,213 | 2,196 | 320.9 | 11 | 27.3 | ND | ND | ND | Strongly Dominated |
| mt-sDNA 50-75, 2 | 7,634 | 2,277 | 319.3 | 12 | 28.1 | ND | ND | ND | Strongly Dominated |
| FIT 45-75, 1 | 19,182 | 2,285 | 340.7 | 12 | 28.9 | 522.1 | 29.8 | 17.5 | Near Efficient |
| mt-sDNA 45-80, 3 | 7,856 | 2,313 | 331.5 | 12 | 28.9 | ND | ND | ND | Strongly Dominated |
| HSgFOBT 50-75, 1 | 13,337 | 2,388 | 320.6 | 12 | 28.2 | ND | ND | ND | Strongly Dominated |
| **FIT 45-80, 1** | **20,958** | **2,405** | **351.1** | **13** | **30.5** | **642.3** | **40.2** | **16.0** | **Efficient** |
| mt-sDNA 50-80, 2 | 8,644 | 2,442 | 330.1 | 13 | 29.9 | ND | ND | ND | Strongly Dominated |
| HSgFOBT 50-80, 1 | 14,792 | 2,544 | 330.0 | 13 | 29.7 | ND | ND | ND | Strongly Dominated |
| mt-sDNA 45-75, 2 | 9,382 | 2,599 | 344.3 | 12 | 29.4 | ND | ND | ND | Strongly Dominated |
| HSgFOBT 45-75, 1 | 16,023 | 2,707 | 343.6 | 12 | 29.2 | ND | ND | ND | Strongly Dominated |
| mt-sDNA 45-80, 2 | 10,046 | 2,712 | 352.8 | 13 | 30.7 | 306.6 | 1.7 | 180.3 | Efficient |
| HSgFOBT 45-80, 1 | 17,489 | 2,859 | 352.9 | 13 | 30.7 | 146.9 | 0.1 | 1093.6 | Efficient |

COL, colonoscopy; CRC, colorectal cancer; FIT, fecal immunochemical test; HSgFOBT, high-sensitivity guaiac-based fecal occult blood test; LYG, life-years gained; mt-sDNA, multitarget stool DNA test; ND, indicates an efficiency ratio is not defined because the strategy is not efficient or near-efficient.
