# Supplementary material for: Estimating the impact of differential adherence on the comparative effectiveness of stool-based colorectal cancer screening using the CRC-AIM microsimulation model
Source: PLoS One. 2020 Dec 29;15(12):e0244431. doi: 10.1371/journal.pone.0244431 (PMC7771985; doi:10.1371/journal.pone.0244431)
Supplement: S5 Table — Results are ordered by total colonoscopies. Results shown are per 1000 individuals free of diagnosed colorectal cancer receiving biennial or triennial mt-sDNA, annual or biennial FIT, and annual or biennial HSgFOBT. (DOCX) [file pone.0244431.s012.docx]

**S5 Table. Incremental efficiency ratios for number of colonoscopies at a fixed screening window of 50–75 or 45–75 assuming perfect (100%) adherence, base-case imperfect adherence rates of 40% FIT vs 34% HSgFOBT vs 70% mt-sDNA, adherence of 50% FIT vs 43% HSgFOBT vs 70% mt-sDNA, or adherence of 60% FIT vs 52% HSgFOBT vs 70% mt-sDNA.** Results are ordered by total colonoscopies. Results shown are per 1000 individuals free of diagnosed colorectal cancer receiving biennial or triennial mt-sDNA, annual or biennial FIT, and annual or biennial HSgFOBT.

| **Screen Window** | **Adherence Scenario** | **Strategies** | **Total COLs** | **LYG** | **ΔCOL** | **ΔLYG** | **Efficiency Ratio**  **(ΔCOL/ΔLYG)** | **Detail** |
| --- | --- | --- | --- | --- | --- | --- | --- | --- |
| 50–75 | 100% FIT/HSgFOBT/mt-sDNA | FIT 50-75, 2 | 1,480 | 273.5 | -- | -- | -- | Efficient |
|  |  | HSgFOBT 50-75, 2 | 1,758 | 278.5 | ND | ND | ND | Weakly Dominated |
|  |  | mt-sDNA 50-75, 3 | 1,957 | 300.0 | ND | ND | ND | Weakly Dominated |
|  |  | FIT 50-75, 1 | 2,037 | 318.1 | 557.0 | 44.6 | 12.5 | Efficient |
|  |  | mt-sDNA 50-75, 2 | 2,277 | 319.3 | 239.9 | 1.2 | 196.6 | Near Efficient |
|  |  | HSgFOBT 50-75, 1 | 2,388 | 320.6 | 351.2 | 2.5 | 142.8 | Efficient |
| 50–75 | 40% FIT/34% HSgFOBT/70% mt-sDNA | FIT 50-75, 2 | 1,056 | 210.9 | -- | -- | -- | Efficient |
|  |  | HSgFOBT 50-75, 2 | 1,160 | 203.6 | ND | ND | ND | Strongly Dominated |
|  |  | FIT 50-75, 1 | 1,269 | 239.4 | 213.4 | 28.5 | 7.5 | Efficient |
|  |  | HSgFOBT 50-75, 1 | 1,371 | 227.4 | ND | ND | ND | Strongly Dominated |
|  |  | mt-sDNA 50-75, 3 | 1,813 | 285.1 | 543.8 | 45.7 | 11.9 | Efficient |
|  |  | mt-sDNA 50-75, 2 | 2,096 | 307.2 | 282.8 | 22.1 | 12.8 | Efficient |
| 50–75 | 50% FIT/43% HSgFOBT/70% mt-sDNA | FIT 50-75, 2 | 1,164 | 229.3 | -- | -- | -- | Efficient |
|  |  | HSgFOBT 50-75, 2 | 1,298 | 223.3 | ND | ND | ND | Strongly Dominated |
|  |  | FIT 50-75, 1 | 1,444 | 263.0 | 280.0 | 33.8 | 8.3 | Efficient |
|  |  | HSgFOBT 50-75, 1 | 1,574 | 252.2 | ND | ND | ND | Strongly Dominated |
|  |  | mt-sDNA 50-75, 3 | 1,813 | 285.1 | 368.6 | 22.1 | 16.7 | Near Efficient |
|  |  | mt-sDNA 50-75, 2 | 2,096 | 307.2 | 651.4 | 44.2 | 14.7 | Efficient |
| 50–75 | 60% FIT/52% HSgFOBT/70% mt-sDNA | FIT 50-75, 2 | 1,256 | 243.0 | -- | -- | -- | Efficient |
|  |  | HSgFOBT 50-75, 2 | 1,408 | 237.3 | ND | ND | ND | Strongly Dominated |
|  |  | FIT 50-75, 1 | 1,594 | 279.5 | 338.5 | 36.5 | 9.3 | Efficient |
|  |  | HSgFOBT 50-75, 1 | 1,747 | 271.4 | ND | ND | ND | Strongly Dominated |
|  |  | mt-sDNA 50-75, 3 | 1,813 | 285.1 | ND | ND | ND | Weakly Dominated |
|  |  | mt-sDNA 50-75, 2 | 2,096 | 307.2 | 501.7 | 27.7 | 18.1 | Efficient |
| 45–75 | 100% FIT/HSgFOBT/mt-sDNA | FIT 45-75, 2 | 1,686 | 300.2 | -- | -- | -- | Efficient |
|  |  | HSgFOBT 45-75, 2 | 2,025 | 305.8 | ND | ND | ND | Weakly Dominated |
|  |  | mt-sDNA 45-75, 3 | 2,196 | 320.9 | ND | ND | ND | Weakly Dominated |
|  |  | FIT 45-75, 1 | 2,285 | 340.7 | 599 | 40 | 14.8 | Efficient |
|  |  | mt-sDNA 45-75, 2 | 2,599 | 344.3 | 314 | 4 | 86.0 | Efficient |
|  |  | HSgFOBT 45-75, 1 | 2,707 | 343.6 | ND | ND | ND | Strongly Dominated |
| 45–75 | 40% FIT/34% HSgFOBT/70% mt-sDNA | FIT 45-75, 2 | 1,190 | 232.9 | -- | -- | -- | Efficient |
|  |  | HSgFOBT 45-75, 2 | 1,325 | 224.3 | ND | ND | ND | Strongly Dominated |
|  |  | FIT 45-75, 1 | 1,433 | 262.7 | 242.9 | 29.8 | 8.1 | Efficient |
|  |  | HSgFOBT 45-75, 1 | 1,564 | 251.7 | ND | ND | ND | Strongly Dominated |
|  |  | mt-sDNA 45-75, 3 | 2,043 | 309.0 | 610.5 | 46.3 | 13.2 | Efficient |
|  |  | mt-sDNA 45-75, 2 | 2,362 | 331.2 | 318.6 | 22.2 | 14.4 | Efficient |
| 45–75 | 50% FIT/43% HSgFOBT/70% mt-sDNA | FIT 45-75, 2 | 1,314 | 251.6 | -- | -- | -- | Efficient |
|  |  | HSgFOBT 45-75, 2 | 1,479 | 245.7 | ND | ND | ND | Strongly Dominated |
|  |  | FIT 45-75, 1 | 1,628 | 286.5 | 313.8 | 34.9 | 9.0 | Efficient |
|  |  | HSgFOBT 45-75, 1 | 1,792 | 278.1 | ND | ND | ND | Strongly Dominated |
|  |  | mt-sDNA 45-75, 3 | 2,043 | 309.0 | 415.5 | 22.6 | 18.4 | Near Efficient |
|  |  | mt-sDNA 45-75, 2 | 2,362 | 331.2 | 734.1 | 44.7 | 16.4 | Efficient |
| 45–75 | 60% FIT/52% HSgFOBT/70% mt-sDNA | FIT 45-75, 2 | 1,413 | 266.7 | -- | -- | -- | Efficient |
|  |  | HSgFOBT 45-75, 2 | 1,603 | 260.8 | ND | ND | ND | Strongly Dominated |
|  |  | FIT 45-75, 1 | 1,795 | 304.3 | 382.0 | 37.6 | 10.2 | Efficient |
|  |  | HSgFOBT 45-75, 1 | 1,987 | 295.8 | ND | ND | ND | Strongly Dominated |
|  |  | mt-sDNA 45-75, 3 | 2,043 | 309.0 | ND | ND | ND | Weakly Dominated |
|  |  | mt-sDNA 45-75, 2 | 2,362 | 331.2 | 567.0 | 27.0 | 21.0 | Efficient |

COL, colonoscopy; CRC, colorectal cancer; FIT, fecal immunochemical test; HSgFOBT, high-sensitivity guaiac-based fecal occult blood test; LYG, life-years gained; mt-sDNA, multitarget stool DNA test; ND, indicates an efficiency ratio is not defined because the strategy is not efficient or near-efficient.
