# Supplementary material for: Estimating the impact of differential adherence on the comparative effectiveness of stool-based colorectal cancer screening using the CRC-AIM microsimulation model
Source: PLoS One. 2020 Dec 29;15(12):e0244431. doi: 10.1371/journal.pone.0244431 (PMC7771985; doi:10.1371/journal.pone.0244431)
Supplement: S8 Table — Results are ordered by patient hours. Results shown are per 1000 individuals free of diagnosed colorectal cancer receiving biennial or triennial mt-sDNA, annual or biennial FIT, and annual or biennial HSgFOBT. (DOCX) [file pone.0244431.s015.docx]

**S8 Table. Incremental efficiency ratios for patient hours related to the screening process at a fixed screening window of 50–75 or 45–75 assuming perfect (100%) adherence, base-case imperfect adherence rates of 40% FIT vs 34% HSgFOBT vs 70% mt-sDNA, 50% FIT vs 43% HSgFOBT vs 70% mt-sDNA adherence, or 60% FIT vs 52% HSgFOBT vs 70% mt-sDNA adherence.** Results are ordered by patient hours. Results shown are per 1000 individuals free of diagnosed colorectal cancer receiving biennial or triennial mt-sDNA, annual or biennial FIT, and annual or biennial HSgFOBT.

| **Screen Window** | **Adherence Scenario** | **Strategies** | **Patient Hours** | **LYG** | **ΔHours** | **ΔLYG** | **Efficiency Ratio**  **(ΔHours/ΔLYG)** | **Detail** |
| --- | --- | --- | --- | --- | --- | --- | --- | --- |
| 50–75 | 100% FIT/HSgFOBT/mt-sDNA | FIT 50-75, 2 | 34,175 | 273.5 | -- | -- | -- | Efficient |
|  |  | mt-sDNA 50-75, 3 | 38,613 | 300.0 | 4,438.6 | 26.4 | 167.9 | Efficient |
|  |  | mt-sDNA 50-75, 2 | 45,388 | 319.3 | 6,774.4 | 19.4 | 350.0 | Efficient |
|  |  | HSgFOBT 50-75, 2 | 46,489 | 278.5 | ND | ND | ND | Strongly Dominated |
|  |  | FIT 50-75, 1 | 49,719 | 318.1 | ND | ND | ND | Strongly Dominated |
|  |  | HSgFOBT 50-75, 1 | 66,216 | 320.6 | 20,828.6 | 1.2 | 16,807.5 | Efficient |
| 50–75 | 40% FIT/34% HSgFOBT/70% mt-sDNA | FIT 50-75, 2 | 23,604 | 210.9 | -- | -- | -- | Efficient |
|  |  | FIT 50-75, 1 | 28,930 | 239.4 | 5,325.2 | 28.5 | 186.6 | Near Efficient |
|  |  | HSgFOBT 50-75, 2 | 29,517 | 203.6 | ND | ND | ND | Strongly Dominated |
|  |  | HSgFOBT 50-75, 1 | 35,449 | 227.4 | ND | ND | ND | Strongly Dominated |
|  |  | mt-sDNA 50-75, 3 | 35,555 | 285.1 | 11,950.6 | 74.2 | 161.1 | Efficient |
|  |  | mt-sDNA 50-75, 2 | 41,531 | 307.2 | 5,976.0 | 22.1 | 269.8 | Efficient |
| 50–75 | 50% FIT/43% HSgFOBT/70% mt-sDNA | FIT 50-75, 2 | 26,277 | 229.3 | -- | -- | -- | Efficient |
|  |  | HSgFOBT 50-75, 2 | 33,287 | 223.3 | ND | ND | ND | Strongly Dominated |
|  |  | FIT 50-75, 1 | 33,394 | 263.0 | ND | ND | ND | Weakly Dominated |
|  |  | mt-sDNA 50-75, 3 | 35,555 | 285.1 | 9,278.0 | 55.8 | 166.2 | Efficient |
|  |  | HSgFOBT 50-75, 1 | 41,203 | 252.2 | ND | ND | ND | Strongly Dominated |
|  |  | mt-sDNA 50-75, 2 | 41,531 | 307.2 | 5,976.0 | 22.1 | 269.8 | Efficient |
| 50–75 | 60% FIT/52% HSgFOBT/70% mt-sDNA | FIT 50-75, 2 | 28,514 | 243.0 | -- | -- | -- | Efficient |
|  |  | mt-sDNA 50-75, 3 | 35,555 | 285.1 | 7,041.0 | 42.1 | 167.3 | Efficient |
|  |  | HSgFOBT 50-75, 2 | 36,402 | 237.3 | ND | ND | ND | Strongly Dominated |
|  |  | FIT 50-75, 1 | 37,331 | 279.5 | ND | ND | ND | Strongly Dominated |
|  |  | mt-sDNA 50-75, 2 | 41,531 | 307.2 | 5,976.0 | 22.1 | 269.8 | Efficient |
|  |  | HSgFOBT 50-75, 1 | 46,304 | 271.4 | ND | ND | ND | Strongly Dominated |
| 45–75 | 100% FIT/HSgFOBT/mt-sDNA | FIT 45-75, 2 | 39,751 | 300.2 | -- | -- | -- | Efficient |
|  |  | mt-sDNA 45-75, 3 | 43,579 | 320.9 | 3,828.5 | 20.7 | 185.3 | Efficient |
|  |  | mt-sDNA 45-75, 2 | 52,318 | 344.3 | 8,738.9 | 23.5 | 372.4 | Efficient |
|  |  | HSgFOBT 45-75, 2 | 54,756 | 305.8 | ND | ND | ND | Strongly Dominated |
|  |  | FIT 45-75, 1 | 57,047 | 340.7 | ND | ND | ND | Strongly Dominated |
|  |  | HSgFOBT 45-75, 1 | 76,709 | 343.6 | ND | ND | ND | Strongly Dominated |
| 45–75 | 40% FIT/34% HSgFOBT/70% mt-sDNA | FIT 45-75, 2 | 26,968 | 232.9 | -- | -- | -- | Efficient |
|  |  | FIT 45-75, 1 | 33,189 | 262.7 | ND | ND | ND | Weakly Dominated |
|  |  | HSgFOBT 45-75, 2 | 34,269 | 224.3 | ND | ND | ND | Strongly Dominated |
|  |  | mt-sDNA 45-75, 3 | 40,350 | 309.0 | 13,382.2 | 76.1 | 175.8 | Efficient |
|  |  | HSgFOBT 45-75, 1 | 41,161 | 251.7 | ND | ND | ND | Strongly Dominated |
|  |  | mt-sDNA 45-75, 2 | 47,162 | 331.2 | 6,812.5 | 22.2 | 307.3 | Efficient |
| 45–75 | 50% FIT/43% HSgFOBT/70% mt-sDNA | FIT 45-75, 2 | 30,064 | 251.6 | -- | -- | -- | Efficient |
|  |  | FIT 45-75, 1 | 38,289 | 286.5 | ND | ND | ND | Weakly Dominated |
|  |  | HSgFOBT 45-75, 2 | 38,586 | 245.7 | ND | ND | ND | Strongly Dominated |
|  |  | mt-sDNA 45-75, 3 | 40,350 | 309.0 | 10,286.4 | 57.5 | 179.0 | Efficient |
|  |  | mt-sDNA 45-75, 2 | 47,162 | 331.2 | 6,812.5 | 22.2 | 307.3 | Efficient |
|  |  | HSgFOBT 45-75, 1 | 47,792 | 278.1 | ND | ND | ND | Strongly Dominated |
| 45–75 | 60% FIT/52% HSgFOBT/70% mt-sDNA | FIT 45-75, 2 | 32,586 | 266.7 | -- | -- | -- | Efficient |
|  |  | mt-sDNA 45-75, 3 | 40,350 | 309.0 | 7,763.5 | 42.4 | 183.4 | Efficient |
|  |  | HSgFOBT 45-75, 2 | 42,172 | 260.8 | ND | ND | ND | Strongly Dominated |
|  |  | FIT 45-75, 1 | 42,817 | 304.3 | ND | ND | ND | Strongly Dominated |
|  |  | mt-sDNA 45-75, 2 | 47,162 | 331.2 | 6,812.5 | 22.2 | 307.3 | Efficient |
|  |  | HSgFOBT 45-75, 1 | 53,694 | 295.8 | ND | ND | ND | Strongly Dominated |

COL, colonoscopy; CRC, colorectal cancer; FIT, fecal immunochemical test; HSgFOBT, high-sensitivity guaiac-based fecal occult blood test; LYG, life-years gained; mt-sDNA, multitarget stool DNA test; ND, indicates an efficiency ratio is not defined because the strategy is not efficient or near-efficient.
