# Supplementary material for: Estimating the impact of differential adherence on the comparative effectiveness of stool-based colorectal cancer screening using the CRC-AIM microsimulation model
Source: PLoS One. 2020 Dec 29;15(12):e0244431. doi: 10.1371/journal.pone.0244431 (PMC7771985; doi:10.1371/journal.pone.0244431)
Supplement: S9 Table — Individuals were randomly assigned numbers of mt-sDNA (max = 11 triennial tests during the screening window) or FIT (max = 31 annual tests during the screening window). (DOCX) [file pone.0244431.s016.docx]

**S9 Table. Predicted outcomes per 1000 individuals screened from ages 45–75 compared with no screening**. Individuals were randomly assigned numbers of mt-sDNA (max=11 triennial tests during the screening window) or FIT (max=31 annual tests during the screening window).

| **Screening strategy** | **Randomly Assigned Number of Tests (n/N, %)** | **Total Stool Tests** | **Total  COLs** | **CRC  Cases** | **CRC  Deaths** | **LY with CRC** | **LYG** | **Incremental COL/**  **Incremental LYG vs FIT** | **Incidence Reduction** | **Mortality Reduction** |
| --- | --- | --- | --- | --- | --- | --- | --- | --- | --- | --- |
| mt-sDNA, 45-75 | Up to 1 (1/11, 9%) | 831 | 421 | 67.8 | 29.5 | 600.1 | 69.6 | 6.5 | 15.6% | 19.4% |
| FIT, 45-75 | Up to 1 (1/31, 3%) | 825 | 245 | 73.8 | 32.5 | 631.1 | 42.4 |  | 8.1% | 11.2% |
| mt-sDNA, 45-75 | Up to 5 (5/11, 45%) | 3,555 | 1,336 | 41.0 | 15.8 | 442.6 | 222.9 | 7.9 | 48.9% | 56.8% |
| FIT, 45-75 | Up to 5 (5/31, 16%) | 3,790 | 746 | 55.6 | 22.3 | 558.8 | 148.0 |  | 30.7% | 39.2% |
| mt-sDNA, 45-75 | Up to 11 (11/11, 100%) | 7,119 | 2,182 | 26.0 | 9.2 | 289.3 | 320.7 | 10.2 | 67.7% | 74.9% |
| FIT, 45-75 | Up to 11 (11/31, 35%) | 7,658 | 1,254 | 41.0 | 15.2 | 464.0 | 229.8 |  | 49.0% | 58.5% |

COL, colonoscopy; CRC, colorectal cancer; FIT, fecal immunochemical test; LY, life-years; LYG, life-years gained; mt-sDNA, multitarget stool DNA test.
