# Supplementary material for: Estimating the impact of differential adherence on the comparative effectiveness of stool-based colorectal cancer screening using the CRC-AIM microsimulation model
Source: PLoS One. 2020 Dec 29;15(12):e0244431. doi: 10.1371/journal.pone.0244431 (PMC7771985; doi:10.1371/journal.pone.0244431)
Supplement: S10 Table — (DOCX) [file pone.0244431.s017.docx]

**S10 Table.** **Deep-C (clinicaltrials.gov identifier, NCT01397747) sensitivity analysis for screening outcomes per 1000 individuals by adherence rate for triennial mt-sDNA and annual FIT in individuals free of diagnosed colorectal cancer at age 40 and screened between ages 50–75 years or 45–75 years.**

| **Screening Strategy and Adherence Rate** | **Stool Tests** | **Follow-up COLs** | **Surveillance  COLs** | **Total  COLs** | **CRC  Cases** | **CRC  Deaths** | **LY with CRC** | **LYG** | **Incidence Reduction** | **Mortality Reduction** |
| --- | --- | --- | --- | --- | --- | --- | --- | --- | --- | --- |
| No screening | 0 | 0 | 0 | 80 | 80.3 | 36.6 | 646.0 | 0.0 | 0.0% | 0.0% |
| mt-sDNA 50-75, 3 |  |  |  |  |  |  |  |  |  |  |
| 10% | 1,801 | 248 | 468 | 765 | 56.1 | 23.8 | 515.5 | 140.9 | 30.2% | 35.0% |
| 20% | 2,968 | 387 | 700 | 1,123 | 45.2 | 18.3 | 448.2 | 204.0 | 43.7% | 50.1% |
| 30% | 3,795 | 478 | 834 | 1,341 | 39.3 | 15.4 | 406.3 | 237.9 | 51.1% | 58.0% |
| 40% | 4,415 | 541 | 921 | 1,487 | 35.7 | 13.6 | 381.5 | 258.5 | 55.6% | 62.8% |
| 50% | 4,889 | 589 | 983 | 1,595 | 33.3 | 12.5 | 362.2 | 272.1 | 58.5% | 65.8% |
| 60% | 5,268 | 628 | 1,027 | 1,676 | 31.6 | 11.7 | 350.1 | 282.4 | 60.6% | 68.1% |
| 70% | 5,578 | 658 | 1,058 | 1,736 | 30.5 | 11.2 | 341.1 | 288.1 | 62.1% | 69.4% |
| 80% | 5,840 | 684 | 1,087 | 1,790 | 29.4 | 10.8 | 330.2 | 293.9 | 63.4% | 70.6% |
| 90% | 6,082 | 708 | 1,108 | 1,834 | 28.5 | 10.3 | 325.2 | 299.2 | 64.5% | 71.8% |
| 100% | 6,290 | 732 | 1,123 | 1,872 | 27.8 | 10.0 | 319.6 | 303.8 | 65.3% | 72.8% |
| FIT 50-75, 1 |  |  |  |  |  |  |  |  |  |  |
| 10% | 2,182 | 138 | 302 | 497 | 64.3 | 27.6 | 572.3 | 101.9 | 19.9% | 24.6% |
| 20% | 4,183 | 243 | 510 | 797 | 53.9 | 22.0 | 518.4 | 166.1 | 32.9% | 40.0% |
| 30% | 6,049 | 331 | 666 | 1,032 | 46.6 | 18.2 | 475.9 | 210.7 | 42.0% | 50.4% |
| 40% | 7,792 | 408 | 787 | 1,223 | 41.2 | 15.5 | 440.6 | 241.0 | 48.7% | 57.6% |
| 50% | 9,432 | 475 | 883 | 1,383 | 37.1 | 13.6 | 408.2 | 262.1 | 53.8% | 62.8% |
| 60% | 10,982 | 537 | 963 | 1,521 | 33.9 | 12.2 | 384.7 | 280.0 | 57.7% | 66.7% |
| 70% | 12,456 | 593 | 1,029 | 1,641 | 31.4 | 11.1 | 362.8 | 291.6 | 60.9% | 69.6% |
| 80% | 13,873 | 645 | 1,085 | 1,748 | 29.2 | 10.2 | 343.7 | 302.2 | 63.6% | 72.1% |
| 90% | 15,226 | 692 | 1,133 | 1,842 | 27.5 | 9.6 | 326.3 | 309.1 | 65.7% | 73.8% |
| 100% | 16,510 | 737 | 1,175 | 1,927 | 26.2 | 9.1 | 314.0 | 315.0 | 67.4% | 75.2% |
| mt-sDNA 45-75, 3 |  |  |  |  |  |  |  |  |  |  |
| 10% | 2,172 | 274 | 537 | 858 | 53.7 | 22.7 | 487.6 | 159.3 | 33.2% | 38.0% |
| 20% | 3,588 | 430 | 791 | 1,255 | 42.3 | 17.0 | 411.8 | 226.8 | 47.3% | 53.6% |
| 30% | 4,585 | 532 | 934 | 1,492 | 36.4 | 14.1 | 367.9 | 261.8 | 54.7% | 61.4% |
| 40% | 5,332 | 603 | 1,026 | 1,653 | 32.8 | 12.4 | 339.2 | 282.6 | 59.2% | 66.0% |
| 50% | 5,913 | 656 | 1,089 | 1,766 | 30.4 | 11.3 | 319.7 | 295.7 | 62.2% | 69.1% |
| 60% | 6,370 | 699 | 1,133 | 1,851 | 28.8 | 10.6 | 305.3 | 304.9 | 64.1% | 71.0% |
| 70% | 6,748 | 734 | 1,169 | 1,920 | 27.6 | 10.1 | 294.7 | 312.2 | 65.6% | 72.5% |
| 80% | 7,063 | 759 | 1,195 | 1,971 | 26.7 | 9.7 | 287.2 | 317.3 | 66.8% | 73.6% |
| 90% | 7,314 | 782 | 1,218 | 2,017 | 26.1 | 9.4 | 280.2 | 320.8 | 67.5% | 74.4% |
| 100% | 7,576 | 814 | 1,240 | 2,070 | 25.2 | 9.0 | 273.8 | 324.7 | 68.7% | 75.5% |
| FIT 45-75, 1 |  |  |  |  |  |  |  |  |  |  |
| 10% | 2,640 | 152 | 348 | 556 | 62.5 | 26.8 | 553.3 | 114.3 | 22.1% | 26.7% |
| 20% | 5,073 | 271 | 583 | 895 | 51.3 | 20.9 | 488.6 | 185.3 | 36.1% | 43.1% |
| 30% | 7,339 | 371 | 753 | 1,155 | 43.7 | 16.9 | 440.0 | 232.3 | 45.6% | 53.8% |
| 40% | 9,470 | 455 | 882 | 1,364 | 38.3 | 14.3 | 400.0 | 263.4 | 52.3% | 60.9% |
| 50% | 11,484 | 530 | 988 | 1,540 | 34.1 | 12.4 | 365.2 | 286.2 | 57.6% | 66.1% |
| 60% | 13,388 | 600 | 1,070 | 1,689 | 30.9 | 11.0 | 338.3 | 303.8 | 61.5% | 70.0% |
| 70% | 15,193 | 664 | 1,140 | 1,822 | 28.5 | 10.0 | 316.0 | 315.5 | 64.5% | 72.7% |
| 80% | 16,953 | 718 | 1,199 | 1,933 | 26.5 | 9.2 | 296.6 | 325.6 | 67.1% | 75.0% |
| 90% | 18,599 | 775 | 1,249 | 2,039 | 24.7 | 8.5 | 278.3 | 333.3 | 69.3% | 76.8% |
| 100% | 20,175 | 827 | 1,293 | 2,135 | 23.4 | 8.0 | 264.4 | 338.6 | 70.9% | 78.1% |

COL, colonoscopy; CRC, colorectal cancer; FIT, fecal immunochemical test; LY, life-years; LYG, life-years gained; mt-sDNA, multitarget stool DNA test.
