# Supplementary material for: Estimating the impact of differential adherence on the comparative effectiveness of stool-based colorectal cancer screening using the CRC-AIM microsimulation model
Source: PLoS One. 2020 Dec 29;15(12):e0244431. doi: 10.1371/journal.pone.0244431 (PMC7771985; doi:10.1371/journal.pone.0244431)
Supplement: S11 Table — Individuals were randomly assigned numbers of mt-sDNA (max = 9 triennial tests during the screening window) or FIT (max = 26 annual tests during the screening window). (DOCX) [file pone.0244431.s018.docx]

**S11 Table. Deep-C (clinicaltrials.gov identifier, NCT01397747) sensitivity analysis of predicted outcomes per 1000 individuals screened from ages 50–75 compared with no screening.** Individuals were randomly assigned numbers of mt-sDNA (max=9 triennial tests during the screening window) or FIT (max=26 annual tests during the screening window).

| **Screening strategy** | **Randomly Assigned Number of Tests (n/N, %)** | **Total Stool Tests** | **Total  COLs** | **CRC  Cases** | **CRC  Deaths** | **LY with CRC** | **LYG** | **Incremental COL/**  **Incremental LYG vs FIT** | **Incidence Reduction** | **Mortality Reduction** |
| --- | --- | --- | --- | --- | --- | --- | --- | --- | --- | --- |
| mt-sDNA, 50-75 | Up to 1 (1/9, 11%) | 819 | 440 | 66.6 | 28.8 | 597.1 | 73.9 | 6.2 | 17.1% | 21.3% |
| FIT, 50-75 | Up to 1 (1/26, 4%) | 813 | 257 | 73.0 | 32.1 | 628.2 | 44.4 |  | 9.1% | 12.3% |
| mt-sDNA, 50-75 | Up to 5 (5/9, 56%) | 3,527 | 1,303 | 39.8 | 15.3 | 438.7 | 223.7 | 7.2 | 50.4% | 58.2% |
| FIT, 50-75 | Up to 5 (5/26, 19%) | 3,702 | 743 | 55.1 | 22.1 | 557.1 | 145.6 |  | 31.5% | 39.8% |
| mt-sDNA, 50-75 | Up to 9 (9/9, 100%) | 6,057 | 1,832 | 28.5 | 10.2 | 327.1 | 298.5 | 7.9 | 64.5% | 72.0% |
| FIT, 50-75 | Up to 9 (9/26, 35%) | 6,255 | 1,072 | 45.0 | 17.1 | 497.4 | 201.8 |  | 44.0% | 53.4% |

COL, colonoscopy; CRC, colorectal cancer; FIT, fecal immunochemical test; LY, life-years; LYG, life-years gained; mt-sDNA, multitarget stool DNA test.
