# Supplementary material for: Estimating the impact of differential adherence on the comparative effectiveness of stool-based colorectal cancer screening using the CRC-AIM microsimulation model
Source: PLoS One. 2020 Dec 29;15(12):e0244431. doi: 10.1371/journal.pone.0244431 (PMC7771985; doi:10.1371/journal.pone.0244431)
Supplement: S1 Appendix — (DOCX) [file pone.0244431.s019.docx]

S1 Appendix

*Microsimulation model*

**Methods**

Adenomas within an individual are generated using a non-homogenous Poisson process based on sex, age, and person-specific risk and assigned a location in either the colon or rectum.[1, 2] Adenomas growth rates based on location are assumed to follow a non-linear growth curve. The cumulative probability of an adenoma transitioning to preclinical CRC is assumed to be a function of adenoma size, age at adenoma initiation, sex, and location of the adenoma. After the adenoma transitions to preclinical CRC, the preclinical CRC is assigned an initial size of 0.5 mm, the sojourn time is determined, and the size of the preclinical CRC upon reaching sojourn time is sampled from a Surveillance, Epidemiology, and End Results (SEER) distribution of CRC sizes. The model assumes simple exponential growth of the CRC. Cancer stage at diagnosis and detection is determined based on CRC size, and the stage of CRC at detection is further determined using a multinomial logistic regression model.[1, 2] Sex-specific cohort life tables from the years 1900-2010 are used for the all-cause (non-CRC) mortality rates. CRC stage-specific survival is based on parametric regression models developed from SEER data for cancers diagnosed from 2000-2003.

For the CRC-AIM screening component it is assumed that CRC screening facilitates the detection and removal of adenomas and preclinical lesions and that the ability of a lesion to be detected is dependent on the sensitivity and reach of the screening test.[3, 4] Screening frequency and adherence rates, as well as the sensitivity and specificity of the screening test, impact the effectiveness of the test. The sensitivity inputs for stool-based tests are per person and are based on the characteristics of the most advanced lesion. The sensitivity inputs for structural tests are per lesion and potential detection of lesions depend on the reach of the test. False positives can occur. Complications (e.g., serious and non-serious gastrointestinal events, cardiovascular events) due to polypectomies can arise and are part of the screening component and the formulas to generate these age-based complications can be found elsewhere.[3] It’s assumed that the only harms from screening are from a colonoscopy with polypectomy, and could be for screening, follow-up, or surveillance, or for the diagnosis of a symptomatic cancer. It was assumed there was no risk of harms from stool-based tests or bowel preparation.[5] The risks of colonoscopy complications are from an analysis by van Hees et al.,[6] that extended the work of Warren et al.[7] In those studies, colonoscopy without polypectomy was not associated with an excess risk of complications when compared with a matched control group without colonoscopy.

The model assumes that in all cases a follow-up colonoscopy occurs after any positive non-colonoscopy screening test.[3, 4] After a negative follow-up colonoscopy, individuals return to their original non-colonoscopy screening test and the next screening is due in 10 years. After a positive follow-up colonoscopy, individuals enter a surveillance colonoscopy period where the next colonoscopy is based on the findings of the latest colonoscopy and continues until at least age 85. Individuals with preclinical lesions that become symptomatic based on sojourn time expiration receive a diagnostic colonoscopy.

**References**

1. CISNET Colorectal Cancer Collaborators. RAND Corporation (CRC-SPIN), 2015. HI.001.03112015.70373. National Cancer Institute Cancer Intervention and Surveillance Modeling Network. 2015. <https://cisnet.cancer.gov/colorectal/profiles.html>. Accessed November 21 2019.

2. Rutter CM, Miglioretti DL, Savarino JE. Bayesian Calibration of Microsimulation Models. J Am Stat Assoc. 2009;104(488): 1338-50. doi:10.1198/jasa.2009.ap07466.

3. Knudsen AB, Zauber AG, Rutter CM, Naber SK, Doria-Rose VP, Pabiniak C et al. Estimation of Benefits, Burden, and Harms of Colorectal Cancer Screening Strategies: Modeling Study for the US Preventive Services Task Force. Jama. 2016;315(23): 2595-609. doi:10.1001/jama.2016.6828.

4. Zauber AG, Knudsen AB, Rutter C, Lansdorp-Vogelaar I, Kuntz KM. Evaluating the benefits and harms of colorectal cancer screening strategies: A collaborative modeling approach. In: AHRQ Technology Assessments. Agency for Healthcare Research and Quality, Rockville, MD. 2015. <https://www.uspreventiveservicestaskforce.org/Home/GetFile/1/16540/cisnet-draft-modeling-report/pdf>.

5. Lin JS, Piper MA, Perdue LA, Rutter C, Webber EM, O'Connor E et al. Screening for Colorectal Cancer: A Systematic Review for the U.S. Preventive Services Task Force. Evidence Synthesis No. 135. AHRQ Publication No. 14-05203-EF-1. Rockville (MD): Agency for Healthcare Research and Quality2016.

6. van Hees F, Zauber AG, Klabunde CN, Goede SL, Lansdorp-Vogelaar I, van Ballegooijen M. The appropriateness of more intensive colonoscopy screening than recommended in Medicare beneficiaries: a modeling study. JAMA Intern Med. 2014;174(10): 1568-76. doi:10.1001/jamainternmed.2014.3889.

7. Warren JL, Klabunde CN, Mariotto AB, Meekins A, Topor M, Brown ML et al. Adverse events after outpatient colonoscopy in the Medicare population. Ann Intern Med. 2009;150(12): 849-57, W152. doi:10.7326/0003-4819-150-12-200906160-00008.
