# Supplementary material for: Estimating the impact of differential adherence on the comparative effectiveness of stool-based colorectal cancer screening using the CRC-AIM microsimulation model
Source: PLoS One. 2020 Dec 29;15(12):e0244431. doi: 10.1371/journal.pone.0244431 (PMC7771985; doi:10.1371/journal.pone.0244431)
Supplement: S2 Appendix — (DOCX) [file pone.0244431.s020.docx]

S2 Appendix

*Cross-Model Validation*

[Medical writing support was provided by David K Edwards V, PhD (Exact Sciences, Madison, WI).]

**Overview and Summary of Outcomes**

We conducted two experiments to demonstrate model cross-validity between CRC-AIM and the CISNET models: (1) regression analyses, in which key outputs from all models were graphically compared for all possible screening strategies; and (2) medical decision-making analyses, in which the final recommended screening strategies for stool testing from all models were compared. We followed the same screening assumptions and method for identifying model-recommendable strategies employed by the CISNET models. These comparisons demonstrated considerable cross-validity across all models; the variability between CRC-AIM and the CISNET models was essentially equivalent to the variability among the CISNET models themselves.

**Experiment 1 Method**

Using the screening outcomes from CRC-AIM[8] and the CISNET models,[9, 3] we performed a set of regressions and a medical decision-making analysis using CRC-AIM. We used period life tables for other-cause mortality instead of the model’s default cohort life table method, aligning with CISNET. For each stool-based screening modality (mt-sDNA, FIT, HSgFOBT), we graphically compared total colonoscopies, life-years gained, CRC incidence reduction, and CRC mortality reduction for each model to an arbitrarily chosen comparator/reference model (SimCRC). Each regression included all 27 screen strategies, consisting of all combinations of screening start/stop ages and screen interval for a given modality.

**Experiment 2 Method**

The medical-decision making analysis consisted of comparing our optimal strategy-selection results for stool screening tests to those of the CISNET models, while remaining as faithful as possible to the assumptions and decision-making process employed by CISNET.[10, 3] We used period life tables for other-cause mortality instead of the model’s default cohort life table method, aligning with CISNET. Like CISNET, we removed 45 as a screening start age, generated efficient frontiers, and only evaluated results within the 50-75 screening window. According to CISNET, the model-recommendable strategy must have LYG within 90% of the benchmark COL, be efficient or near-efficient within its screening class, and have an efficiency ratio at or below the slope threshold set by the benchmark COL. If all these criteria are met, the recommendable strategy for a given model and screening class is the one that offers the most LYG. There can be no strategies selected for a given screening class. The final set of model recommendable strategies from CISNET included those recommendable by at least 2 of the models. We compared the selected stool screening strategy, the efficiency ratio of the selected strategy, and %LYG of the benchmark COL for all four models.

**Experiment 1 Results**

For each stool-screening modality, the regression plots demonstrate CRC-AIM was comparable to the CISNET models with regards to total COL, LYG, CRC incidence reduction and CRC mortality reduction (**Fig B1-B3**).

**Figure B1. mt-sDNA strategies.**


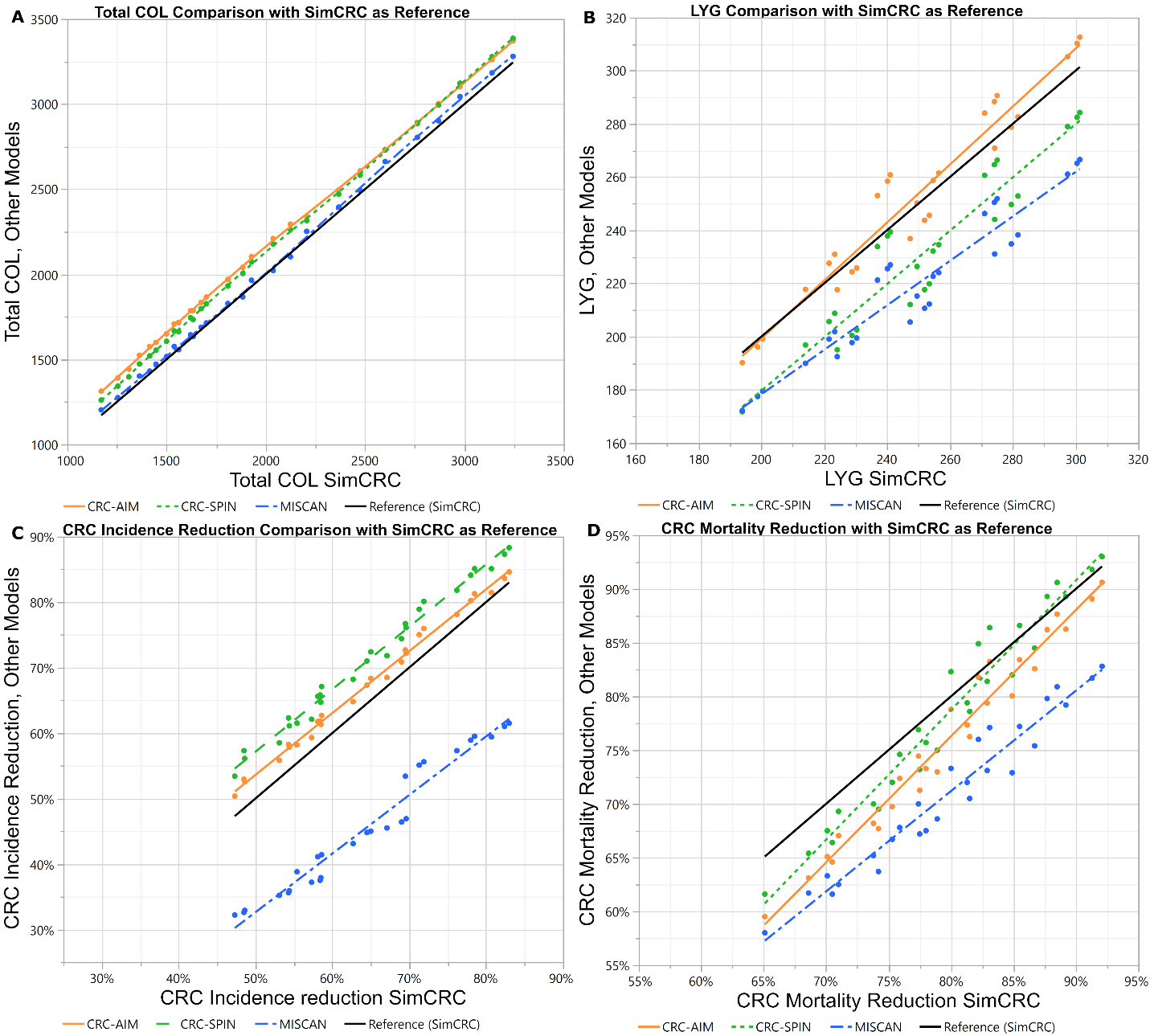


**Figure B2. FIT strategies.**

**
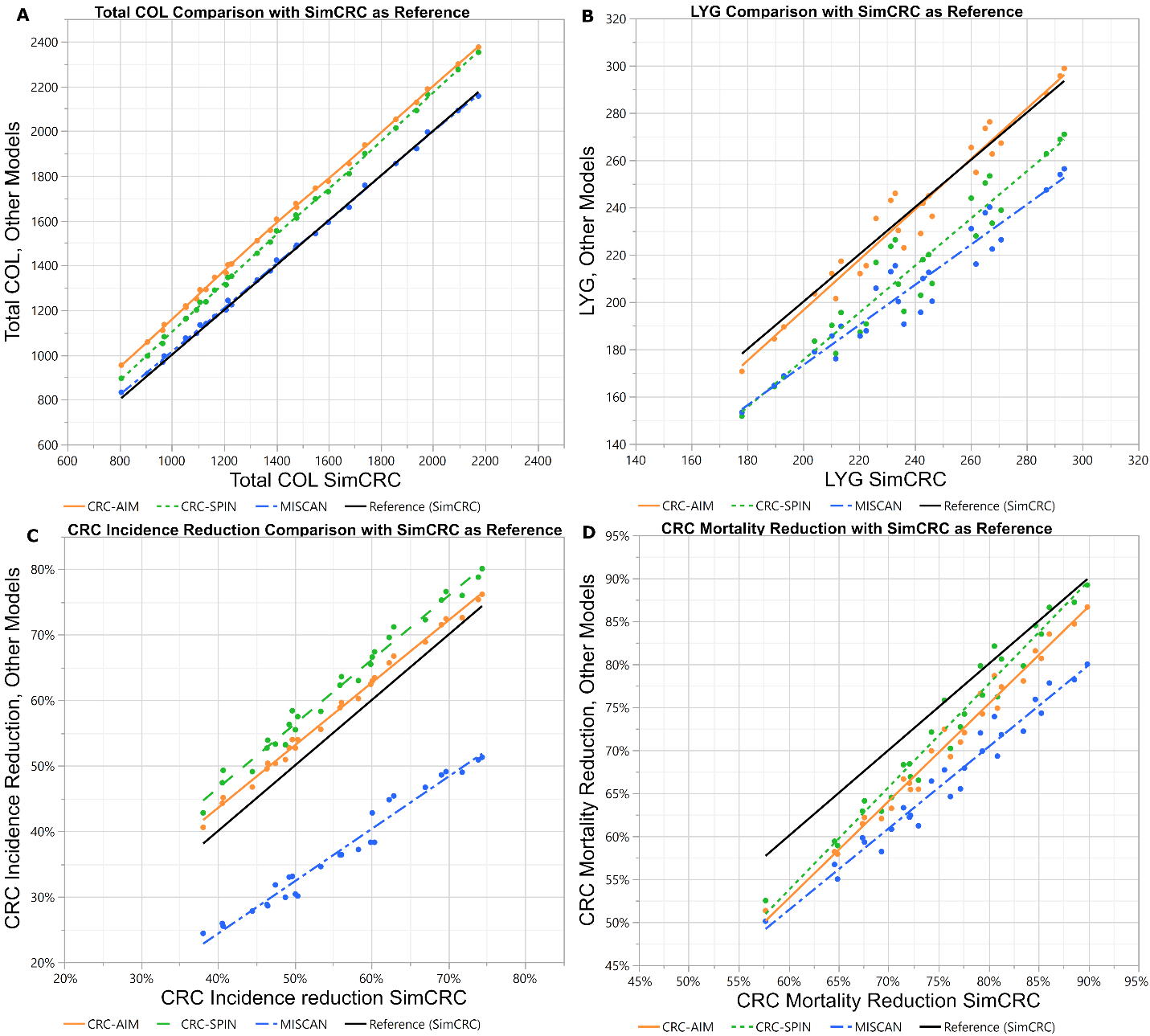
**

**Figure B3. HSgFOBT strategies.**

**
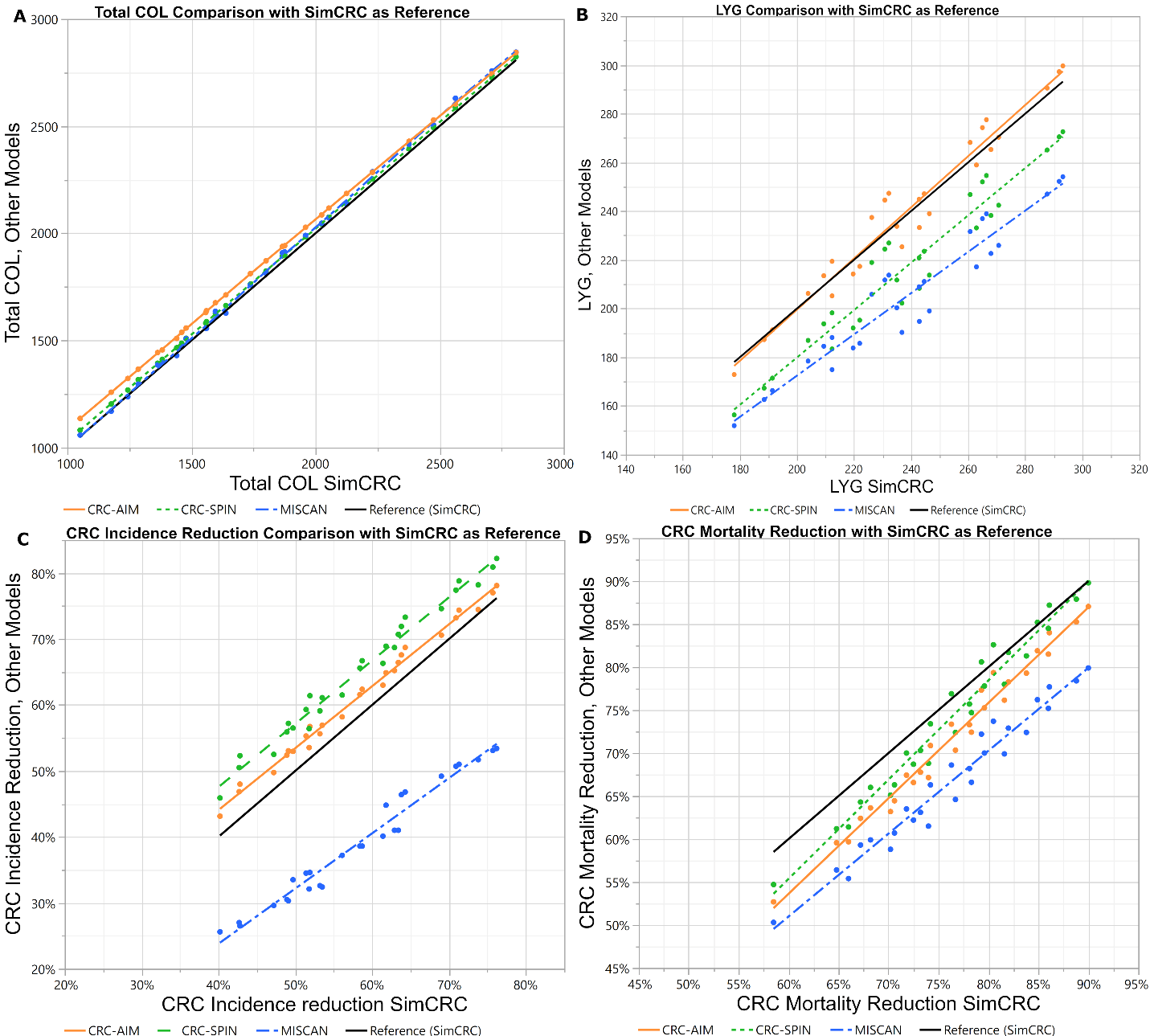
**

**Experiment 2 Results**

Model outcomes pertaining to optimal strategy selection, based on harm/benefit tradeoffs, are provided in **Table B1.** CRC-AIM generates the same benchmark COL strategy as other models. The benchmark COL efficiency ratio generated by CRC-AIM is within the range of the three CISNET models. If perfect adherence is assumed, CRC-AIM also selects the same optimal stool screening strategy as the three CISNET models, with an efficiency ratio and percent LYG of colonoscopy within the range of three CISNET models.

**Table B1. Comparison of stool test model-recommended strategy outcomes between CRC-AIM and the CISNET models (MISCAN, SimCRC, and CRC-SPIN).** Screening window described as a range from screening start age to stop age (years), with screening interval in years.

|  | **CRC-AIM** | **MISCAN** | **SimCRC** | **CRC-SPIN** |
| --- | --- | --- | --- | --- |
| **Benchmark COL**  interval, ER slope threshold | 50-75, 10y  Threshold ER = 52 | 50-75, 10y  Threshold ER = 39 | 50-75, 10y  Threshold ER = 55 | 50-75, 10y  Threshold ER = 65 |
|  | **Optimal strategy (interval, ER, %LYG)** | | | |
| Stool test (FIT, HSgFOBT, mt-sDNA) | FIT: 50-75, 1y  ER = 19  %LYG = 90.5 | FIT: 50-75, 1y  ER = 21  %LYG = 93.1 | FIT 50-75, 1yr  ER = 24  %LYG = 94.5 | FIT 50-75, 1y  ER = 17  %LYG = 90.4 |

ER, efficiency ratio; FIT, fecal immunochemical test; HSgFOBT, high-sensitivity guaiac-based fecal occult blood test; %LYG, life-years gained of benchmark colonoscopy; mt-sDNA, multitarget stool DNA test.

**References**

1. CISNET Colorectal Cancer Collaborators. RAND Corporation (CRC-SPIN), 2015. HI.001.03112015.70373. National Cancer Institute Cancer Intervention and Surveillance Modeling Network. 2015. <https://cisnet.cancer.gov/colorectal/profiles.html>. Accessed November 21 2019.

2. Rutter CM, Miglioretti DL, Savarino JE. Bayesian Calibration of Microsimulation Models. J Am Stat Assoc. 2009;104(488): 1338-50. doi:10.1198/jasa.2009.ap07466.

3. Knudsen AB, Zauber AG, Rutter CM, Naber SK, Doria-Rose VP, Pabiniak C et al. Estimation of Benefits, Burden, and Harms of Colorectal Cancer Screening Strategies: Modeling Study for the US Preventive Services Task Force. Jama. 2016;315(23): 2595-609. doi:10.1001/jama.2016.6828.

4. Zauber AG, Knudsen AB, Rutter C, Lansdorp-Vogelaar I, Kuntz KM. Evaluating the benefits and harms of colorectal cancer screening strategies: A collaborative modeling approach. In: AHRQ Technology Assessments. Agency for Healthcare Research and Quality, Rockville, MD. 2015. <https://www.uspreventiveservicestaskforce.org/Home/GetFile/1/16540/cisnet-draft-modeling-report/pdf>.

5. Lin JS, Piper MA, Perdue LA, Rutter C, Webber EM, O'Connor E et al. Screening for Colorectal Cancer: A Systematic Review for the U.S. Preventive Services Task Force. Evidence Synthesis No. 135. AHRQ Publication No. 14-05203-EF-1. Rockville (MD): Agency for Healthcare Research and Quality2016.

6. van Hees F, Zauber AG, Klabunde CN, Goede SL, Lansdorp-Vogelaar I, van Ballegooijen M. The appropriateness of more intensive colonoscopy screening than recommended in Medicare beneficiaries: a modeling study. JAMA Intern Med. 2014;174(10): 1568-76. doi:10.1001/jamainternmed.2014.3889.

7. Warren JL, Klabunde CN, Mariotto AB, Meekins A, Topor M, Brown ML et al. Adverse events after outpatient colonoscopy in the Medicare population. Ann Intern Med. 2009;150(12): 849-57, W152. doi:10.7326/0003-4819-150-12-200906160-00008.

8. Description and validation of the novel Colorectal Cancer and Adenoma Incidence & Mortality (CRC-AIM) Microsimulation model. [database on the Internet]2020. Available from: <https://www.biorxiv.org/content/10.1101/2020.03.02.966838v1>. Accessed: 03/05/2020

9. Zauber AG, Knudsen AB, Carolyn R, Naber SK, Doria-Rose P, Pabiniak C et al. 178 Evaluating the Benefits and Harms of Colorectal Cancer Screening Strategies: A Collaborative Modeling Approach to Inform the US Preventive Services Task Force. Gastroenterology. 2016;150(4). doi:10.1016/s0016-5085(16)30279-7.

10. Zauber A, Knudsen AB, Rutter CM, Lansdorp-Vogelaar I, Kuntz KM. Technical Report: Evaluating the benefits and harms of colorectal cancer screening strategies: a collaborative modeling approach. 2015. <https://www.uspreventiveservicestaskforce.org/Home/GetFile/1/16540/cisnet-draft-modeling-report/pdf>.
